# Investigation on Cement-Stabilized Base with Recycled Aggregate and Desert Sand

**DOI:** 10.3390/ma17174262

**Published:** 2024-08-28

**Authors:** Fengchao Liu, Yongjun Qin, Yiheng Yang

**Affiliations:** School of Architecture and Engineering, Xinjiang University, Urumqi 830049, China; ikernylfc@163.com (F.L.); gtr96033@outlook.com (Y.Y.)

**Keywords:** recycled coarse aggregate, desert sand, cement-stabilized macadam, mechanical property, microstructure

## Abstract

This paper mainly explores the feasibility of using desert sand (DS) and recycled aggregate in cement-stabilized bases. Recycled coarse aggregate (RCA) and DS serve as the substitutes of natural coarse and fine aggregates, respectively, in cement-stabilized bases. A four-factor and four-level orthogonal test is designed to analyze the unconfined compressive strength, splitting tensile strength, and compressive resilient modulus. Furthermore, this paper investigates the effects of cement content, fly ash (FA) replacement rate, RCA replacement rate, and DS replacement rate on the road performance of cement-stabilized bases composed of RCA and DS. The test results reveal that the performance of cement-stabilized bases with partial RCA instead of natural coarse aggregate (NCA) and partial DS instead of natural fine aggregate satisfies the road use. The correlation and microscopic analyses of the test results imply the feasibility of applying DS and recycled aggregate to cement-stabilized bases. This paper calculates and evaluates the life cycle of carbon emissions of desert sand and recycled coarse aggregate cement-stabilized macadam (DRCSM) and finds that both DS and RCA can reduce the carbon emissions of CSM, which has a positive effect on improving the environment and solving the climate crisis. It is hoped that this paper can offer a solid theoretical foundation for promoting the application of DS and recycled aggregate in road engineering.

## 1. Introduction

As a basic building material, cement-stabilized macadam (CSM) represents one of the most popular road base materials worldwide [1]. With robust bearing capacity, CSM is composed of suitable graded aggregate, cement with an aggregate mass of 3–15%, and water with optimum water content [2]. Desert sand (DS) functions as the other highway base material with poor material properties, such as loose particles, small cohesion, weak gradation, low natural water content, and poor water permeability [3]. However, how to make good use of the characteristics of DS in road construction in desert areas to achieve the specified base strength becomes a novel direction for its usage [4]. On one hand, maintenance and reconstruction of cement concrete roads will produce a lot of waste concrete. On the other hand, in response to the needs of national infrastructure development, highway projects require a large amount of sand and gravel these years, gradually increasing the demand for sand and gravel resources. Consequently, using recycled aggregates is of great significance [5,6,7,8]. Natural DS is abundant and concentrated in Northwest China, including Xinjiang. However, it is less exploited, leading to the contradiction between supply and demand. If local materials can play a better role in promoting the application of DS in highway engineering, it can not only solve the insufficient medium sand resources but also reduce the transportation costs.

Many experts have conducted extensive research on the application of recycled aggregates in CSM, proving that they can be applied in CSM and have obtained appropriate amounts and gradations of recycled aggregates [9,10,11,12]. Corradini [13] explored the application of recycled aggregate in road pavement engineering through the triaxial test of cyclic load and reported that the recycled aggregate exhibited stable rebound performance before and after cement addition. By measuring the mechanical strength and road performance of recycled CSM, Lyu et al. [14,15] believed that the utilization of recycled aggregate meets the specified requirements for road bases. Zhao et al. [16] demonstrated that maintaining the content of recycled aggregate at 25–30% is conducive to enhancing the fatigue resistance of CSM. Hu et al. [17] unveiled that the skeleton-dense cement-stable base structure can improve the strength and crack resistance of CSM. Zou [18] used cement and fly ash (FA) to prepare recycled aggregate CSM. He proposed that the compressive strength of recycled aggregate was higher than that of natural aggregate, suggesting the potential use of full recycled aggregate in preparation of CSM. Yu [19] prepared CSM by replacing natural aggregate with 0%, 25%, 50%, 75%, and 100% recycled aggregate at 3%, 4%, 5%, and 6% cement content, respectively, and obtained the optimum content of recycled aggregate and cement through the mechanical index of the mixture. Lan et al. [20] evaluated the mechanical properties and shrinkage properties of cement stabilized base using 0–2.36 mm recycled fine aggregate (RFA). The results showed that the effect of 0–2.36 mm RFA on the mechanical properties of CSM was closely related to the cement content and had a certain degree of deterioration on the shrinkage properties of CSM. Arul et al. [21] applied recycled aggregate and recycled glass (FRG) together in CSM and found that the 7-day unconfined compressive strength of the base met the local minimum requirements when the FRG content was 30%, proving that the two can be applied together in CSM.

Employing DS to fill the road base can not only obtain local materials, reduce transportation costs, and solve the problem of lack of sand for engineering, but it can also reduce the problem of desertification. DS is a type of aggregate with poor material properties and poor gradation. Scholars have studied the performance of desert sand cement-stabilized bases, proving that desert sand can be applied to cement-stabilized bases [22,23], and the performance of desert sand cement-stabilized bases can be improved by adding some other admixtures [24,25]. Netterberg et al. [26] conducted a long-term observation on the test section of DS road base in South Africa and found that the road structure can maintain stability. Similarly, Song et al. [27] applied 6% DS instead of fine aggregate to the CSM and demonstrated that it could meet the road performance of the base mixture through the indoor test and test-section-tracking detection. Ma et al. [28] developed an indoor mix-rate test by adding cement and gravel in DS and proved the feasibility of applying DSCSM as a base in a highway.

This paper aims to investigate the road performance of cement-stabilized bases when the recycled aggregate and DS are both applied as the primary materials of the road base and refer to existing research on concrete carbon emissions to analyze the carbon emissions of DRCSM throughout its lifecycle [29,30], providing a theoretical basis for practical application of DS and recycled aggregate, thus promoting their wider usage in road construction.

## 2. Materials and Methods

### 2.1. Testing Materials

(1) Cement: Tianshan P·O42.5R cement produced by Xinjiang Urumqi Tianshan Cement Plant (Urumqi, China), with an actual strength of 44.6 MPa; 

(2) Natural coarse aggregate (NCA): pebbles in Urumqi, Xinjiang produced in Xinjiang Kangsheng Lvyuan Building Materials Co., Ltd. (Urumqi, China), with particle size of 5–30 mm and bulk density of 2687 kg/m^3^;

(3) Recycled coarse aggregate (RCA): it is broken from the abandoned cement stabilized base after the demolition of a military apron in Liugong Town, Changji Prefecture, Xinjiang. The particle size ranges from 5 mm to 30 mm, and the bulk density is 2613 kg/m^3^. The detailed performance indicators are outlined in Table 1.

(4) Fine aggregate: washing-machine-made sand in Xinjiang, with fineness modulus of 3.0 and apparent density of 2487 kg/m^3^;

(5) DS: taken from the Taklimakan Desert, with fineness modulus of 0.12, average particle size of 0.118 (Table 2), bulk density of 1334 kg/m^3^, apparent density of 2790 kg/m^3^, water content of 0.4%, and mud content of 0.4%.

(6) FA: produced in Xinjiang Kangsheng Lvyuan Building Materials Co., Ltd. (Urumqi, China). 

### 2.2. Gradation Design

**Figure 1:**
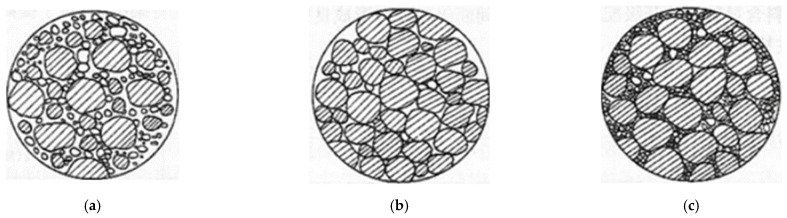
Typical composition and structure of CSM. (**a**) Suspended dense structure; (**b**) skeleton void structure; (**c**) skeleton dense structure.

**Figure 2:**
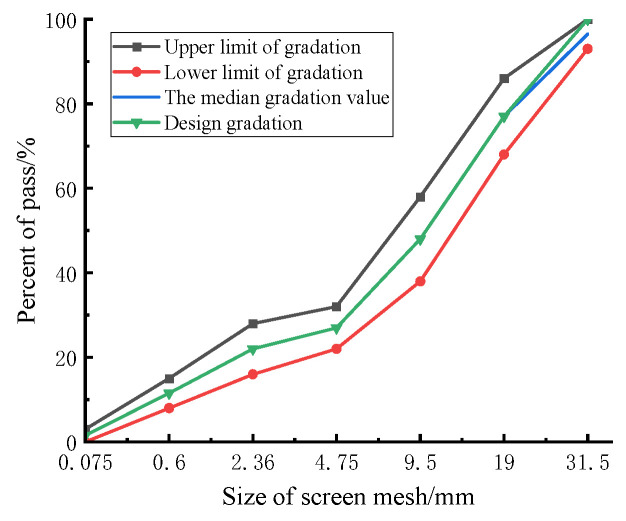
Aggregate grading curve.

To ensure the performance comparability of the CSM composing DS and RCA (denoted as DRCSM herein) under different mix ratios, the gradation curve of each mixture group in the test is adjusted to the same as far as possible. According to the requirements of the specification [31], the grading of aggregate size has its corresponding upper and lower limits, and the upper limit, lower limit, and median value correspond to different internal skeleton structures, as shown in Figure 1. The coarse, median, and fine gradations of the DRCSM correspond to the skeleton pore structure, skeleton dense structure, and suspension dense aggregate, respectively. The internal structure corresponding to the intermediate distribution value shows good strength, so it is designed according to this gradation. Table 3 presents the passing rate of aggregate screening under different sieves, and the corresponding aggregate gradation curve is illustrated in Figure 2.

### 2.3. Experimental Design

Orthogonal test was designed. The factors and levels of orthogonal test and specific mix rate of DRCSM is listed in Table 4 and Table 5. The amounts of water and dry mixture are determined based on the optimum water content and maximum dry density obtained from the test. The specimens for unconfined compressive strength, splitting tensile strength, and anti-scouring test are cylindrical (150 mm × 150 mm), and those for dry shrinkage test are beam (150 mm × 150 mm × 400 mm). The specimens are fabricated by static pressure method using universal press, and the degree of compaction is 98% according to the specification [32]. Also, 5%, 6%, 7%, and 8% P·O32.5R Portland cement are added to the DRCSM. Since the use of RCA and DS at the same time will lead to the potential strength reduction of DRCSM, the replacement rates of the two are fixed at 0%, 25%, 50%, 75%, and 0%, 10%, 20%, and 30%.

### 2.4. Test Methods

**Figure 3:**
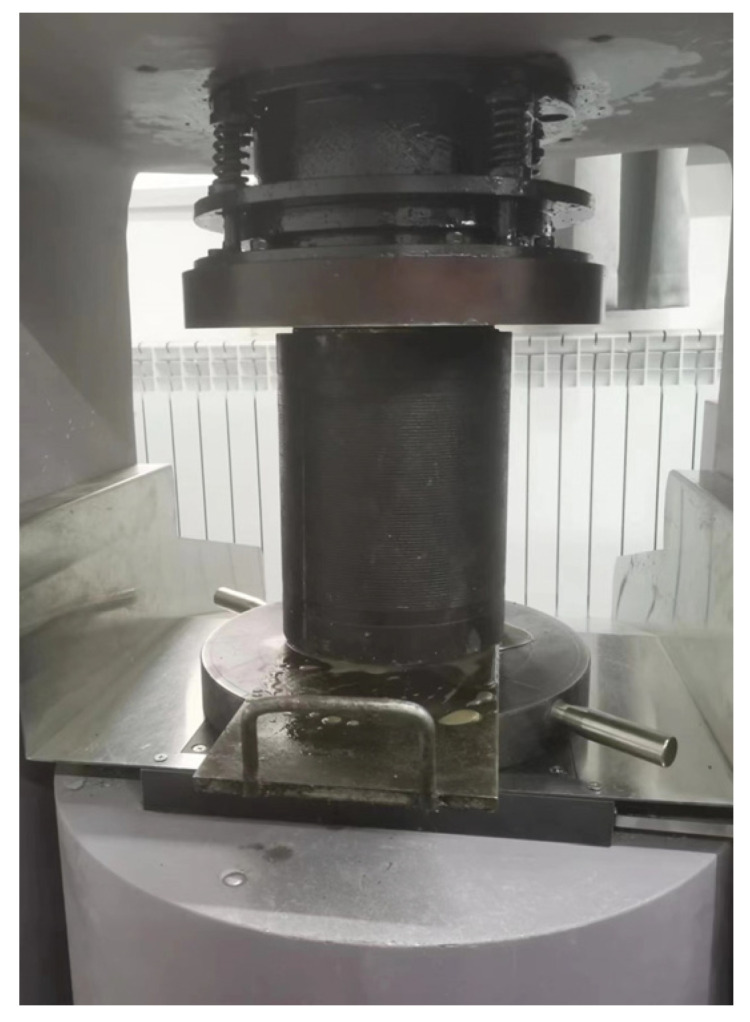
Specimen compaction molding.

Using the results of the standard compaction test, the mass of the mixture required to prepare the specimen is calculated based on the optimum water content and the maximum dry density obtained from the compaction test. The specimen preparation and strength test are conducted in adherence to the standard requirements [32]. The specimen (Φ 150 mm × 150 mm) is formed by static pressure method, as shown in Figure 3. Six specimens are fabricated for each mixture rate and undergo the parallel test in turn, with the average value serving as the test result. After molding, the specimens were demolded and placed in a standard curing room set at a temperature of 20 °C ± 2 °C and a relative humidity of more than 95% for standard curing.

**Figure 4:**
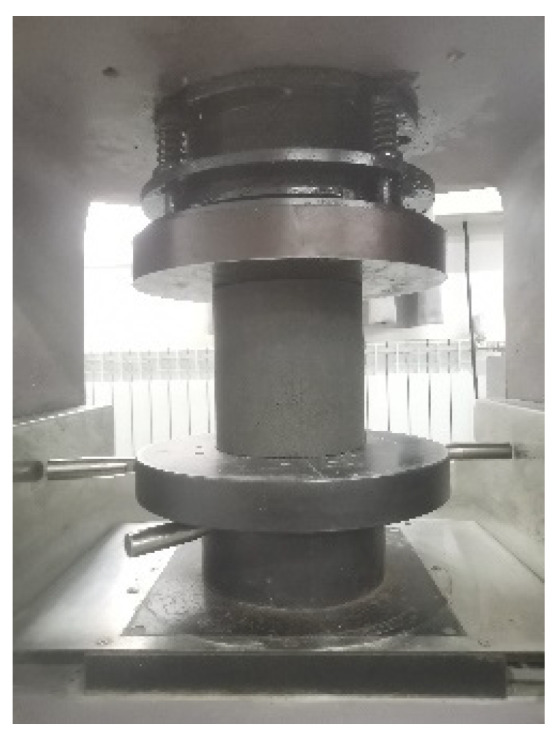
Machine for unconfined compressive strength test.

**Figure 5:**
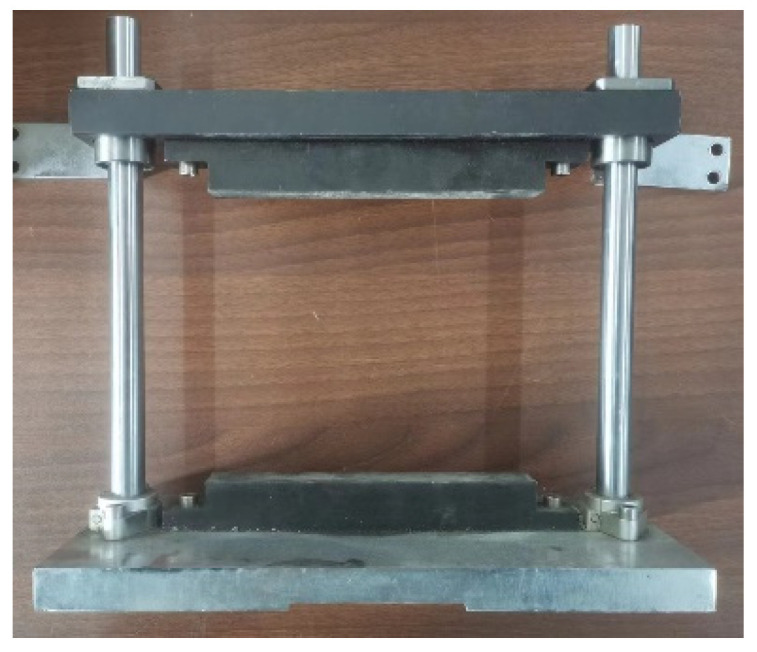
Fixture for splitting test.

The specimens are cured for 7, 28, and 90 d, followed by 24 h of soaking after the specified age. Subsequently, the surface moisture is eliminated, and the mass is weighed. The height is measured to be accurate at 0.1 mm, and the surface is flattened with a scraper. Next, the unconfined compressive strength and splitting tensile strength are tested by universal pressure-testing machine, as displayed in Figure 4, and the fixture used in the splitting test is shown in Figure 5. During the test, the loading rate is set as 1 mm/min, and the pressure when the specimen is cracked is assigned as the maximum pressure.

The compressive strength of the mixture specimen is calculated according to Equation (1):

(1) $$R_{c}=\frac{P}{A}$$

In the equation: $R_{c}$—Compressive strength of the specimen/MPa;

P—Maximum pressure at specimen failure/N;

A—The sectional area of the specimen/mm^2^.

The splitting tensile strength of cement-stabilized base specimen is calculated according to Equation (2):

(2) $$R_{i}=\frac{2P}{\pi dh}\left(sin2\alpha -\frac{a}{d}\right)$$

In the equation: $R_{i}$—Splitting tensile strength of the specimen/MPa; 

d—Diameter of specimen/mm;

a—Width of the layering/mm;

α—The central angle corresponding to the half strip width/°;

P—Maximum pressure at specimen failure/N;

h—Height of the specimen after immersion/mm.

Expression for calculating the compressive resilient modulus under each load level is provided in Equation (3):

(3) $$E_{c}=\frac{ph}{l}$$

In the equation: $E_{c}$—Compressive resilient modulus/MPa;

p—Unit pressure/MPa;

h—Height of the specimen/mm;

l—Rebound deformation of the specimen/mm.

### 2.5. Carbon-Emission Life-Cycle Calculation of Recycled Aggregate

Xiao et al. [29] utilized life-cycle assessment (LCA) methodology to delineate the carbon emissions associated with the utilization of recycled aggregates within road engineering, categorizing them into direct and indirect emissions. Direct emissions primarily comprise CO_2_ emissions stemming from fossil energy utilization across various stages of recycled aggregate production and application, along with those arising from the cement manufacturing process. Indirect emissions encompass CO_2_ emissions arising from energy acquisition processes (such as electricity and diesel usage), constituting an integral component of recycled aggregate carbon emissions.

The total carbon emission $C_{T}$ and LCA-derived carbon emission $C_{L}$ of recycled concrete can be quantitatively computed using Equations (4) and (5).

(4) $$C_{T}=C_{1}+C_{2}+C_{3}+C_{4}+C_{6}$$

(5) $$C_{L}=C_{T}-C_{5}$$

Among these equations, $C_{i}$ represents the carbon emissions of each stage of the life cycle of recycled concrete. Specifically, $C_{1}$ consists of carbon emissions $C_{1a}$ stemming from raw material production, and carbon emissions $C_{1b}$ originating from the transportation of raw materials to the recycled concrete mixing station. Both components can be calculated using Equations (6) and (7).

(6) $$C_{1a}=\sum_{i}\left(\sum_{j}a_{ij}K_{j}\right)m_{i}+g_{1}m_{1}$$

(7) $$C_{1b}=\sum_{i}\left(d^{y}+b_{j}^{y}k_{j}^{\prime }\right)s_{i}m_{i}$$

$C_{2}$ represents the carbon emission incurred during the production process of recycled concrete, which can be determined using Equation (8).

(8) $$C_{2}=\sum_{j}e_{j}K_{j}$$

$C_{3}$ denotes the carbon emission arising from the transportation of ready-mixed recycled concrete to the construction site, ascertainable through Equation (9). It is pertinent to note that the average transportation distance is 30 km.

(9) $$C_{3}=\left(d+b_{j}k_{j}^{\prime }\right)s_{c}M$$

$C_{4}$ denotes the carbon emissions attributed to recycled concrete during the construction phase. It is posited that the carbon emissions of recycled concrete and conventional concrete are substantially equivalent during construction. Drawing from reference [20], an average value of 21.8 is adopted as the carbon emissions of the primary components, representing the carbon emissions of unit recycled concrete construction $C_{4}$. 

$C_{6}$ denotes the carbon emissions incurred during the demolition of recycled concrete. This includes carbon emissions $C_{6a}$ generated throughout the demolition process and carbon emissions $C_{6b}$ arising during the transportation of waste concrete. As per reference [33], precise calculation of carbon emissions during the demolition process poses challenges. Consequently, it is approximated at 90% of the construction process, yielding $C_{6a}={0.9C}_{4}$, while $C_{6b}$ can be calculated using Equation (6).

Due to the varying data requirements across each stage of the life cycle, the variables in Equations (6)–(9) are explained as follows: $m_{i}$ represents the quantity of the first type of raw materials per unit of recycled concrete; $a_{ij}$ denotes the quantity of energy (e.g., electric energy, coal, diesel) consumed in the production of raw materials; $K_{j}$ signifies the carbon emission coefficient of various energy sources, comprising the aggregate of both direct carbon emission coefficient $k_{j}$ and the indirect carbon emission coefficient $k_{j}^{\prime }$. $g_{1}$ represents the carbon emission associated with each material in cement production; d stands for the direct carbon emission coefficient engendered by transportation usage; $b_{j}$ signifies the unit transportation energy consumption for the respective transportation mode; $s_{i}$ represents the transportation distance of raw materials for category *i*; $s_{c}$ represents the transportation distance of recycled concrete; *m* signifies the total mass of recycled concrete per cubic meter.

Xiao et al. [34] discovered that the alkaline substances present in concrete possess the capability to absorb atmospheric CO_2_ and undergo reaction, thereby exerting a compensation effect on the environment. Furthermore, they noted a correlation between the carbonation depth of recycled concrete and the replacement rate of recycled aggregate. To facilitate prediction, they proposed an equation for estimating the carbonation depth of recycled concrete, presented as Equation (10).

(10) $$x_{c}={839g_{RC}\left(1-R\right)}^{1.1}\sqrt{\frac{\left(\frac{W}{\gamma_{c}C}-0.34\right)}{8.03\gamma_{HD}\gamma_{c}C}n_{0}t}$$

$x_{c}$ represents the carbonization depth, serving as an indicator of concrete’s carbonization extent. It is noteworthy that higher carbonization degree in concrete corresponds to increased CO_2_ absorption over time. $C_{5}$ denotes the carbon-absorption capacity resulting from the carbonation of recycled concrete, quantifiable through Equation (11).

(11) $$C_{5}=0.044m_{0}\frac{x_{c}A_{surface}}{1}$$

R signifies relative humidity; W represents the unit water consumption of recycled concrete; $\gamma_{c}$ denotes the correction coefficient of cement type, assumed as 1 within this study; $\gamma_{HD}$ represents the correction coefficient for cement hydration. For curing ages exceeding 90 days, $\gamma_{HD}$ assumes a value of 0.85 at 1 d and 28 d, with linear interpolation for other ages. $n_{0}$ stands for the volume fraction of CO_2_; t signifies the duration of carbonization; and $g_{RC}$ represents the influence coefficient of recycled aggregate, where its value interpolates linearly between 1 and 1.5 across a range of recycled aggregate replacement rates from 0% to 100%. $m_{0}$ denotes the quantity of CO_2_ absorbed per unit of recycled concrete upon complete carbonization, calculated following reference [35]. $A_{surface}$ represents the exposed surface area per unit of recycled concrete, with a recommended value of 5.68 m^2^ based on reference [36].

## 3. Results and Discussion

### 3.1. Unconfined Compression Strength

**Figure 6:**
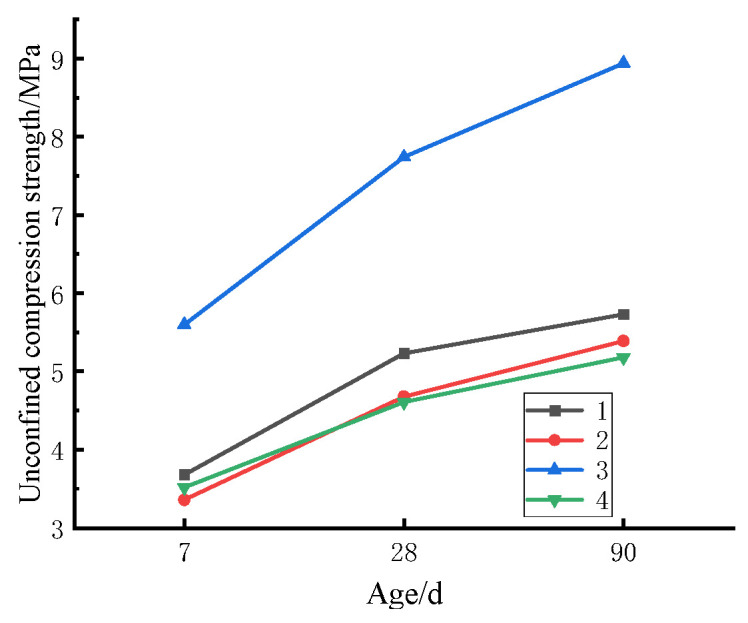
Changes in compressive strength of CSM with curing age.

Table 6 lists the unconfined compressive strength obtained by orthogonal test at 7, 28, and 90 d. Figure 6 explicates the evolution of compressive strength of mixture with age under 6% cement dosage, which is served as an example herein. The figure signifies that the compressive strength of the CSM mixture changes with the curing age. Notably, the growth rate is fast before 28 d but slows down after that day. Consequently, when CSM functions as the semi-rigid road base, the curing time should be maintained until the basic strength is formed to avoid damage to the base caused by the load in the subsequent construction. When the cement content is greater than or equal to 8%, the compressive strength of CSM can satisfy the specification for heavy traffic on the first-class highway.

**Figure 7:**
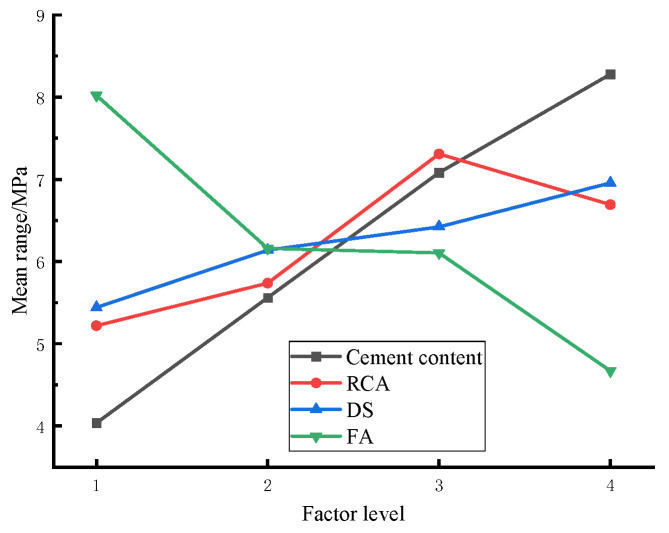
Mean range of each factor at each level.

In the specification [32], the unconfined compressive strength obtained on the 7th day (referred to as 7 d unconfined comprehensive strength) is undertaken as the reference to measure the road performance of the cement-stabilized base, with a requirement of over 4 MPa for special heavy traffic on highways. The specific range analysis on the orthogonal test results is presented in Table 7 and Figure 7.

It is evident from Table 7 that different factors exert varying influences on the 7 d unconfined compressive strength. Specifically, in the selected four-level change interval, the influence of each factor on the 7 d unconfined compressive strength adheres to the following order: cement dosage > FA replacement rate > RCA replacement rate > DS replacement rate. Furthermore, the above figure reveals that the compressive strength of the mixture is higher under the 30% DS replacement rate and 50% recycled aggregate replacement rate.

Range analysis on the test results cannot estimate the test error. On the other hand, variance analysis can identify the differences and error fluctuations among the test results generated by interaction among distinct levels, thus improving the accuracy of the result analysis. Consequently, the reliability of the analysis results can be enhanced by performing the variance analysis on the test results. Table 8 shows the variance analysis of the orthogonal test results of the 7 d unconfined compressive strength.

It becomes evident from Table 8 that the F test results of the four orthogonal factors are:

a. The cement content: F > F_0.01_, proving that the cement content significantly affects the 7-d unconfined compressive strength;
b. The replacement rate of RCA: F_cement_ > F > F_0.01_, indicating that the RCA replacement rate exerts a highly significant effect on the 7 d unconfined compressive strength, which is weaker than that of cement content;
c. The DS replacement rate: F_0.01_ > F > F_0.05_, suggesting that the DS replacement rate greatly influences the 7 d unconfined compressive strength;
d. FA replacement rate: F_cement_ > F > F_RCA_ > F_0.01_, implying that the influence of FA replacement rate on the 7 d unconfined compressive strength is highly significant, showing its degree between cement content and RCA replacement rate.

Based on the results of range analysis and variance analysis, the following order can be concluded when comparing their influence on the unconfined compressive strength of cement-stabilized base: cement content > FA replacement rate > RCA replacement rate > DS replacement rate. It signifies that the appropriate amount of RCA and DS can improve the compressive strength of the CSM mixture.

### 3.2. Splitting Strength

The stipulated 90 d splitting strength of the base is 0.4–0.6 MPa [32]. Range analysis of the orthogonal test results is presented in Table 9. It suggests that the four orthogonal factors influencing the 90 d splitting tensile strength exhibit consistent order with that of the compressive strength. Specifically, in the selected four-level change interval, an order of the influence degree of factors on the 90 d splitting tensile strength is observed as cement dosage > FA replacement rate > RCA replacement rate > DS replacement rate.

The variance analysis on the orthogonal test results of the 90 d splitting tensile strength in Table 10 displays the F test results of the four orthogonal factors, as follows:

a. The cement content: F > F_0.01_, proving that the cement content plays a significant role in influencing the 90 d splitting tensile strength;
b. The RCA replacement rate: F_cement_ > F > F_0.01_, indicating that the RCA replacement rate exerts a highly significant effect on the 90 d splitting tensile strength, which is weaker in contrast to that of cement content;
c. The DS replacement rate: F_0.01_ > F > F_0.05_, signifying that the DS replacement rate significantly influences the 90 d splitting tensile strength;
d. The FA replacement rate: F_cement_ > F > F_RCA_ > F_0.01_, suggesting that the FA replacement rate extremely impact the 90 d splitting tensile strength, whose degree is between the cement content and the RCA replacement rate.

Based on the results of range analysis and variance analysis, it can be concluded that the order of influence degree of the four factors on the unconfined compressive strength of the cement-stabilized base is: cement dosage > FA replacement rate > RCA replacement rate > DS replacement rate. It reveals that increasing the cement content can enhance the binding among mixture particles and the base strength. FA exhibits poor performance, so it can decrease the strength of the base as an alternative material for cement. Meanwhile, FA demonstrates distinct cementitious properties with cement. As a result, it will induce a significant decrease in base strength if too much FA is applied to substitute cement. When the RCA replacement rate is 50%, the optimal effect can be achieved. The analysis unveils that the RCA used in this crushing exhibits better performance, showing little effect in the case of partial use. However, it will still affect the base strength when it is applied to substitute the most natural aggregate. The particle size of DS is smaller. Using DS to partially replace fine aggregate can improve the gradation of the mixture, and an appropriate amount of DS can fill the pores among the aggregates, making the interior of the base denser, thereby improving the strength of the cement-stabilized base.

The 90 d compressive strength and splitting tensile strength of DRCSM in range analysis were taken separately to analyze the correlation between the compressive strength and splitting tensile strength of CSM by curve fitting.

**Figure 8:**
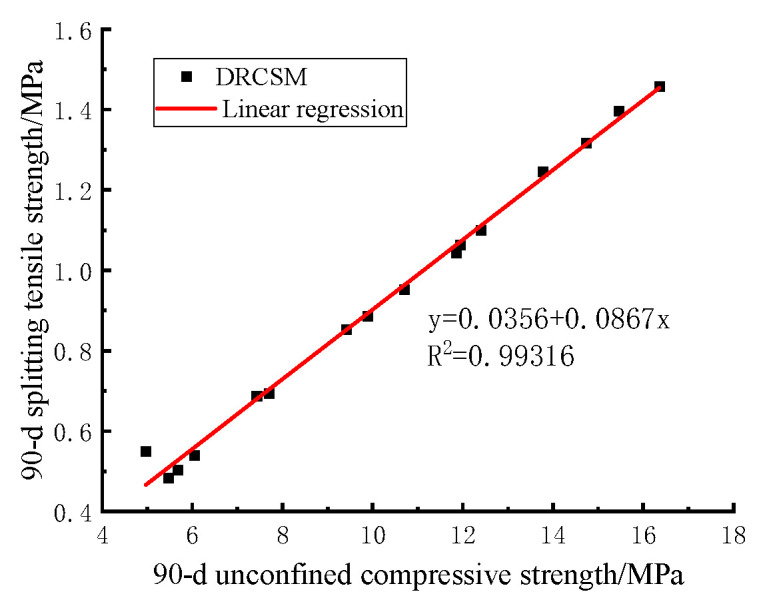
Correlation between 90 d compressive strength and splitting tensile strength of mixture.

Figure 8 reflects that the linear fitting equation of the 90 d compressive strength and splitting tensile strength of DRCSM can be expressed as follows:

(12) $$R_{i}=0.03562+0.08672R_{c}$$

The correlation coefficient is $R^{2}=0.99316$, which suggests a good correlation between the 90 d compressive strength and the splitting tensile strength of the mixture.

In the equation: $R_{i}$—Splitting tensile strength of the specimen/MPa;

$R_{c}$—Compressive strength of the specimen/MPa;

$R^{2}$—Correlation coefficient.

The evaluation parameters of the CSM mixture stipulated in the specification include 7 d unconfined compressive strength and 90 d splitting tensile strength. Due to a similar development law of compressive strength and splitting tensile strength with age in the experimental results, the correlation between 7 d compressive strength and 90 d splitting tensile strength of the mixture can be further analyzed under the controlled test amount.

**Figure 9:**
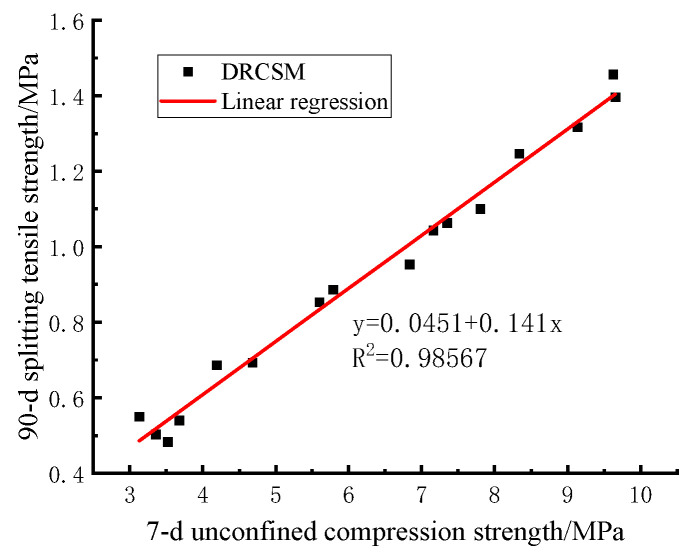
Correlation between 7-d compressive strength and splitting tensile strength of mixture.

It can be observed from Figure 9 that the linear fitting equation of 90 d compressive strength and splitting tensile strength of DRCSM is expressed as follows:

(13) $$R_{i}=0.04513+0.14076R_{c}$$

The correlation coefficient is $R^{2}=0.98567$, implying a good correlation between the 90 d compressive strength and the splitting tensile strength of the mixture. Therefore, the 90 d splitting tensile strength can be estimated according to Equation (13) and the 7 d compressive strength of CSM required by the specification.

### 3.3. Compressive Resilient Modulus

As presented in Table 11, under the influence of four factors, the compressive resilient modulus of the mixture gradually increases with the prolonged curing age. Taking 6% cement content as an example, the 28 d compressive resilient modulus values of the four groups are 1.26 times, 1.19 times, 1.32 times, and 1.18 times of the 7 d values, and the 90 d compressive resilient modulus values are 1.74 times, 1.75 times, 1.75 times, and 1.68 times of the 28 d values. Therefore, the compressive resilient modulus of DRCSM increases with the rise of curing age.

The range analysis of the orthogonal test results for the compressive resilient modulus of the mixture is summarized in Table 12. It signifies that the four orthogonal factors on the 90 d compressive resilient modulus exhibit consistent influence degree with the compressive strength. The selected four-level change interval reveals that the influence degree of the factors on the 90 d splitting tensile strength follows the order of cement dosage > FA replacement rate > RCA replacement rate > DS replacement rate.

Furthermore, Table 13 displays the variance analysis results of the orthogonal test results for the 90 d compressive resilient modulus.

Table 13 indicates that the F test results of the four orthogonal factors are as follows:

a. The cement content: F > F_0.01_, proving the significant effect of cement content on the 90 d compressive resilient modulus;
b. The RCA replacement rate: F < F_0.05_, indicating that the replacement rate of RCA fails to substantially influence the 90 d compressive resilient modulus;
c. The DS replacement rate: F < F_0.05_, showcasing that the DS replacement rate does not significantly affect the 90 d compressive resilient modulus;
d. The FA replacement rate: F_cement_ > F > F_0.01_, indicating that the FA replacement rate plays a highly significant effect in influencing the 90 d compressive resilient modulus, but the degree is less than the cement content.

Analysis on results of range analysis and variance analysis suggests that the order of the four factors when influencing the compressive resilient modulus of the cement-stabilized base is cement content > FA replacement rate > RCA replacement rate > DS replacement rate, with no significant influence of RCA and DS replacement rates. Moreover, with the increase of cement content, the mixture will form more cement during the curing, enhancing the density of internal structure, thus increasing the cohesion, deformation resistance, and compressive resilient modulus of the base. FA possesses poorer mechanical properties than cement, so the base strength will be reduced after substituting cement with FA. Moreover, the mineral impurities such as calcium oxide in the FA will hinder the hydration reaction of the cement, decreasing the amount and quality of cement in the mixture, thereby reducing the compressive rebound modulus of the base.

Analysis in Section 3.2 reveals that if the 90 d compressive resilient modulus can be predicted by the 7 d unconfined compressive strength required by the specification, the test cycle can be greatly reduced, greatly improving the test efficiency. Consequently, the 7 d compressive strength and 90 d compressive resilient modulus of DRCSM were selected to analyze the correlation between them using curve fitting.

**Figure 10:**
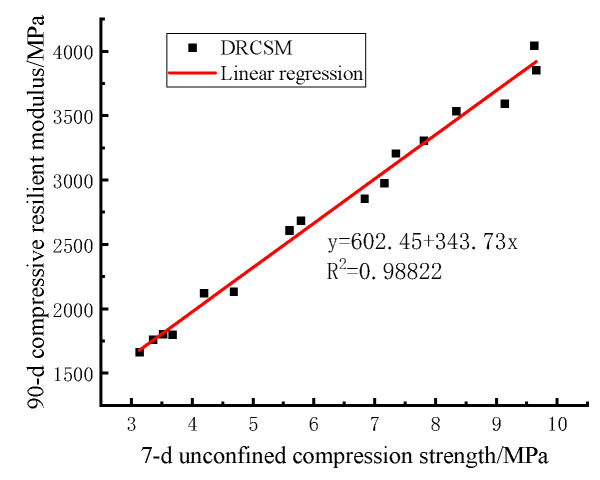
Correlation between the 7 d compressive strength and 90 d compressive resilient modulus of mixture.

As illustrated in Figure 10, the linear fitting equation of the 7 d compressive strength and 90 d compressive rebound modulus of DRCSM can be written as follows:

(14) $$E_{c}=602.45+343.73R_{c}$$

The correlation coefficient is $R^{2}=0.98822$, which means that the 7 d compressive strength of the mixture demonstrates a good correlation with the 90 d compressive rebound modulus. Therefore, the 90 d compressive rebound modulus can be estimated by the 7 d compressive strength of the specimen through Equation (14).

In the equation: $E_{c}$—Compressive resilient modulus of the specimen/MPa;

$R_{c}$—Compressive strength of the specimen/MPa;

$R^{2}$—Correlation coefficient.

The results of variance and range analyses indicate that the compressive resilient modulus of the mixture is primarily affected by the cement content. Table 14 lists the mean values of the 90 d compressive resilient modulus under the influence of the varying cement contents in the variance analysis. After that, the correlation between the cement content and the 90 d compressive resilient modulus is fitted by the curve.

**Figure 11:**
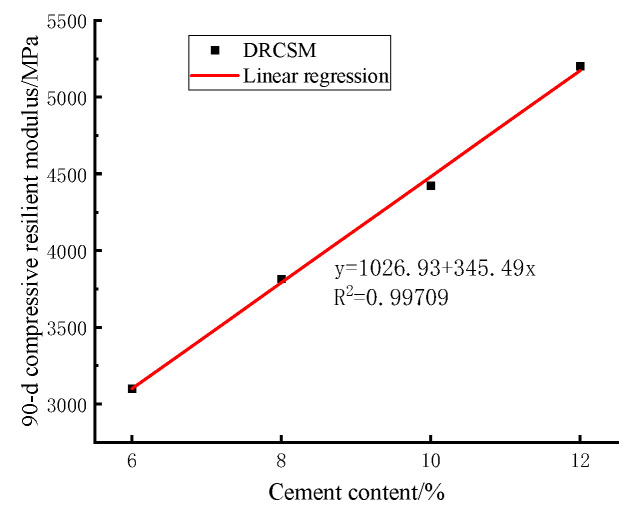
Correlation between cement content and 90 d compressive resilient modulus of mixture.

As explained in Figure 11, the following linear fitting equation is observed between the cement content of DRCSM and the 90 d compressive rebound modulus:

(15) $$E_{c}=1026.93+345.49C$$

In the equation: $E_{c}$ is the compressive resilient modulus of the specimen, expressed in MPa, C is the Cement content in the specimen, expressed in %, and $R^{2}$ is the correlation coefficient.

The correlation coefficient is $R^{2}=0.98822$, suggesting the existence of a good correlation between the cement content of the mixture and the 90 d compressive resilient modulus. Additionally, the above Equation (15) can be adopted to estimate the 90 d compressive resilient modulus under different cement contents.

### 3.4. Microstructures of DRCSM

Building upon the previous section, it can be concluded that introducing a certain amount of DS is beneficial to improve the strength of CSM and the influence of FA on the strength of CSM is highly significant. Furthermore, specimens were sampled from the two materials under different substitution rates to analyze the influence of introducing them on the microscopic properties of CSM using scanning electron microscopy (SEM).

**Figure 12:**
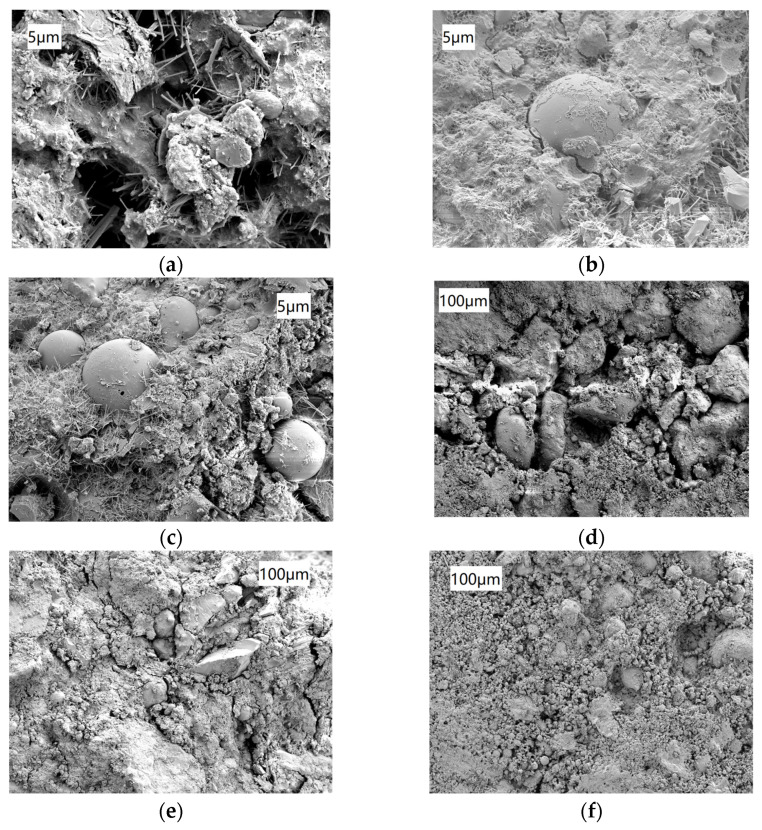
Electron microscope photos CSM with different FA and DS contents. (**a**) DS 30%, FA 0%; (**b**) DS 30%, FA 25%; (**c**) DS 30%, FA 75%; (**d**) DS 0, FA 50%; (**e**) DS 10%, FA 50% and (**f**) DS 30%, FA 50%.

As displayed in Figure 12, with the increase in FA replacement rate, the number of spherical FA particles gradually increases. Large pores are observed in Figure 12a, with obvious columnar ettringite crystals (AFt) growing among pores. In Figure 12b, the microstructure becomes denser, and the aggregate surface is wrapped by floccules, accompanied by the formation of clusters of rod-like products. There are cracks at the interface between FA particles and aggregates. Figure 12c shows that there are more FA particles, and the interface between FA particles and aggregate is well combined without obvious cracks. There are also more C-S-H gels, as well as needle-like and cluster-like Aft crystals. It reflects that the particle fineness of FA is higher, and the incorporation of FA instead of cement can fill the tiny pores in the aggregate, thus improving the compactness and homogeneity of the internal structure of the mixture. Secondly, active substances, such as alumina and silicate contained in FA, can react with cement, generating new hydration products, which is helpful to enhance the bonding performance inside the mixture. Thirdly, higher particle fineness and spherical shape of FA enhance its dispersion and adhesion in the mixture, restricting the development of cracks inside the mixture.

By comparing the SEM images of specimens with different DS replacement rates shown in Figure 12, the following results can be obtained. The CSM in Figure 12d exhibits loose internal structure, more pores among aggregates, and uneven distribution of compounds. With the increase of the DS replacement rate, the compactness of the mixture gradually increases, the number and width of cracks decrease, and the distribution of coarse and fine aggregates is more uniform, as demonstrated in Figure 12e. In Figure 12f, the number of cracks is significantly reduced, the pores among aggregates are composed of numerous fine pores with smaller pores, and the particles are closer. In addition, coarse aggregates are tightly wrapped with cement-hydration products and fine aggregates. These observations suggest that increasing the DS replacement rate makes the mixture denser, thus enhancing its strength. On the one hand, the finer particle size of DS can fill the gaps among coarse aggregates in the mixture, making the pore structure among the skeletons denser or more continuous. On the other hand, the cementation between DS and cement or FA can enhance the bonding performance of aggregates, thereby improving the bearing capacity of the mixture.

**Figure 13:**
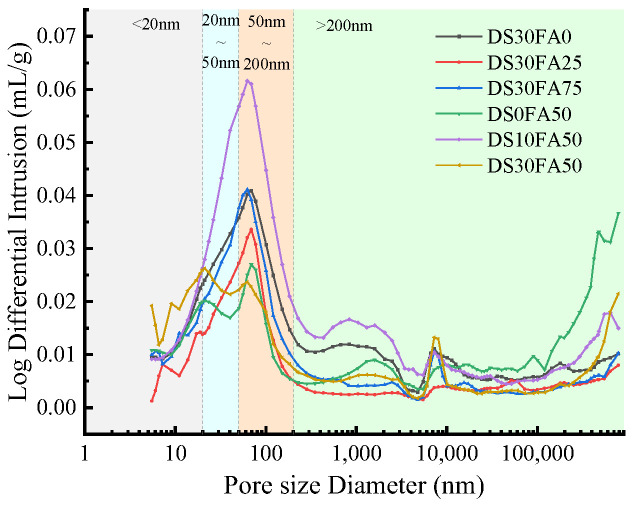
The pore-size distribution inside the specimen under different mix ratios.

Wu [37] proposed to divide the pore size into harmless pores (<20 nm), less harmful pores (~20 nm–50 nm), harmful pores (~50 nm–200 nm), and more harmful pores (>200 nm) according to the impact on the concrete performance, and to increase the pores below 50 nm and reduce the pores above 100 nm can significantly improve the performance of concrete. The distribution and proportion of different pore sizes in the mercury intrusion test are shown in Figure 13 and Table 15.

Further MIP tests shall be conducted on the specimen scanned by electron microscope, and the test results are shown in Figure 13 and Table 15. According to test results, it can be seen that the pore structure of the specimens is better when the substitution rates of desert sand and fly ash are 30% and 50%. The proportion of harmless and less harmful pores in the specimens increases with the increase of desert sand-substitution rate, proving that desert sand can improve the pore structure inside DRCSM, thereby enhancing its mechanical properties. The proportion of harmless pores and less harmful pores in the specimen increases first and then decreases with the increase of the FA replacement rate, reaching a peak at 50%. Analysis suggests that substances such as silicates and aluminates in FA will react with calcium hydroxide and hydrated sulfates in cement, causing the cementitious system of cement to be destroyed, resulting in an insufficiently dense internal structure of DRCSM and an increase in harmful pores. Therefore, it is advisable to control the FA replacement rate to around 50% to ensure that the DRCSM has a good pore structure and mechanical properties inside.

## 4. Calculation and Analysis of Carbon Emissions of Recycled Aggregate

Taking Urumqi as a case study, the carbon emissions associated with recycled cement-stabilized macadam featuring a 6% cement content were calculated and compared with conventional concrete. As per reference [24], the environmental conditions in Urumqi entail a relative humidity of 57.5% and an atmospheric CO_2_ concentration of 0.03%. As elucidated in the previous section, the carbon emissions throughout other stages of the life cycle of recycled concrete are contingent upon the replacement rate of recycled aggregate. This section conducts a comparative analysis, computing the life cycle carbon emissions of cement-stabilized macadam employing natural aggregate and varying recycled aggregate replacement rates of 25%, 50%, 75%, and 100%. Additionally, the calculation encompasses the carbonation depth and CO_2_ absorption of recycled cement-stabilized macadam during the carbonation stage in Urumqi. Assumptions for this analysis include the utilization of diesel trains for transportation, with an average transportation distance of 30 km during the transportation process. The cement-stabilized base undergoes maintenance for 28 days, while the anticipated road-service life spans 30 years. The calculated values for $C_{2}$, $C_{3}$, $C_{4}$, and $C_{6}$ are delineated as follows: $C_{2}$ = 2.4 kg, $C_{3}$ = 7.8 kg, $C_{4}$ = 21.8 kg, and $C_{6}$ = 27.4 kg [20]. The calculation results are shown in Table 16.

The findings presented in Table 16 underscore that the life-cycle carbon emissions associated with recycled CSM, featuring a 6% cement content are notably lower than those of conventional CSM. Across each stage, carbon emissions stemming from raw material production constitute the highest proportion, exhibiting a gradual increase corresponding to the replacement rate of recycled aggregate, ranging from 62.7–71.5%. Specifically, when the replacement rate of recycled aggregate varies from 25%, 50%, 75%, to 100%, the life-cycle carbon emissions of unit-recycled CSM measure 320.5, 316.1, 312.2, and 306.2, respectively. These values represent approximately 98.0%, 96.7%, 95.5%, and 93.6% of the carbon emissions observed in ordinary CSM. Moreover, across each stage of the recycled CSM life cycle, both the carbon emissions from raw material transportation process and the carbonization phase exhibit a declining trend with increasing recycled aggregate-replacement rate. The carbon emissions arising from raw material transportation processes represent 11.2% to 20.1% of the overall life cycle, exhibiting a reduction rate of 9.5% with a decrease in recycled aggregate-replacement rates from 0% to 100%. Additionally, carbon absorption during the carbonization phase accounts for 0.9% to 2.2% of the life cycle. Therefore, optimizing the proximity of recycled aggregate to the mixing station becomes imperative to enhance the environmental advantages of recycled CSM compared to ordinary CSM. This underscores the significance of promoting and utilizing recycled aggregates within cement-stabilized bases in Urumqi, as it demonstrates tangible environmental benefits.

## 5. Conclusions

This paper delved into the application of the mixture of DR and recycled aggregates in CSM. Through crushing, processing, and screening, the waste-concrete-recycled aggregate was prepared, and its basic performance is tested. A four-factor and four-level orthogonal test was designed to investigate the comprehensive performance of DRCSM. In addition, the standard compaction test was completed to understand its mechanical properties. The main conclusions of this paper are as follows:

(1) With the increase of curing age, the DRCSM exhibits consistent development laws (increment) in unconfined compressive strength, splitting tensile strength, and compressive resilient modulus. The DRCSM exhibits a better mechanical property when the cement dosage DS and RCA replacement rate are 30% and 50%, respectively.

(2) In the variance analysis, it is evident that the compressive strength and splitting tensile strength of the mixture show similar results. Cement content and FA replacement rate significantly influence the strength of the mixture, but the influence of RCA and DS replacement rates on the compressive resilient modulus is not remarkable. The TEM analysis reveals that introducing appropriate contents of DS and FA can better the internal microstructure of CSM, improve the internal compactness of CSM, enhance the bonding performance among aggregates, and elevate the bearing capacity of the CSM.

(3) For the mechanical properties of DRCSM, when the cement content is more than 6%, its 7 d unconfined compressive strength and 90 d splitting tensile strength meet the requirements of the first-class highway special heavy traffic in the specification. A good correlation is observed by fitting and drawing the curves for compressive strength, splitting tensile strength, and compressive rebound modulus under the change of cement content. Meanwhile, splitting tensile strength and compressive rebound modulus can be predicted by compressive strength.

(4) Through the calculation and analysis of the life-cycle carbon emissions of DRCSM, it is found that the use of desert sand and recycled coarse aggregate in the cement-stabilized base can help reduce CO_2_ emissions. The way to reduce carbon emissions is mainly reflected in the transportation and production stage of raw materials. It is necessary to control the transportation distance of raw materials, which proves that the popularization and use of desert sand and recycled coarse aggregate in the cement-stabilized base has a certain value in environmental benefits.

## Figures and Tables

**Table 1:** Performance indicators of coarse aggregate.

Indicators	Unit	NCA	RCA	Specification
Apparent specific gravity	g·cm^−3^	2.687	2.613	≥2.50
Water absorption	%	0.97	4.93	≤3.0
Soil content	%	0.8	1.4	≤1.5
Flat, elongated particle content	%	6.4	13.7	≤18
Ruggedness	-	4.8	9.5	≤12
Crush value	%	15.7	21.9	≤26

**Table 2:** Particle-size distribution of DS.

Grain Size	0.16	0.075	<0.075
Cumulative sieve margin/%	11.8	88.2	10.2

**Table 3:** The passing rate of aggregate screening.

Size of Screen Mesh/mm	Passing Rate/%			
	Upper Limit	Lower Limit	Median Gradation	Design Gradation
31.5	100	93	96.5	100
19.0	86	68	77	77
9.5	58	38	48	48
4.75	32	22	27	27
2.36	28	16	22	22
0.6	15	8	11.5	11.5
0.075	3	0	1.5	1.5

**Table 4:** Factors and levels of orthogonal test.

Level	Factor			
	Cement Content	RCA Replacement Rate	DS Replacement Rate	FA Replacement Rate
1	5%	0%	0%	0%
2	6%	25%	10%	25%
3	7%	50%	20%	50%
4	8%	75%	30%	75%

**Table 5:** Specimen mix design.

Specimen Number	Cement Content	RCA Replacement Rate	DS Replacement Rate	FA Replacement Rate
DRCSM-1	5%	0%	0%	0%
DRCSM-2	5%	25%	10%	25%
DRCSM-3	5%	50%	20%	50%
DRCSM-4	5%	75%	30%	75%
DRCSM-5	6%	0%	10%	50%
DRCSM-6	6%	25%	0%	75%
DRCSM-7	6%	50%	30%	0%
DRCSM-8	6%	75%	20%	25%
DRCSM-9	7%	0%	20%	75%
DRCSM-10	7%	25%	30%	50%
DRCSM-11	7%	50%	0%	25%
DRCSM-12	7%	75%	10%	0%
DRCSM-13	8%	0%	30%	25%
DRCSM-14	8%	25%	20%	0%
DRCSM-15	8%	50%	10%	75%
DRCSM-16	8%	75%	0%	50%

**Table 6:** Unconfined compressive strength of mixture 7, 28, and 90 d after curing.

Group Number	Unconfined Compression Strength/MPa		
	7 d	28 d	90 d
DRCSM-1	3.68	5.23	5.73
DRCSM-2	3.36	4.68	5.39
DRCSM-3	5.60	7.74	8.94
DRCSM-4	3.52	4.61	5.18
DRCSM-5	4.19	6.07	7.05
DRCSM-6	3.13	4.24	4.72
DRCSM-7	9.13	12.81	14.00
DRCSM-8	5.79	8.29	9.40
DRCSM-9	4.68	6.41	7.32
DRCSM-10	6.83	9.01	10.17
DRCSM-11	7.16	10.35	11.27
DRCSM-12	9.65	12.92	14.68
DRCSM-13	8.34	11.71	13.09
DRCSM-14	9.62	13.25	15.54
DRCSM-15	7.35	10.03	11.34
DRCSM-16	7.80	10.40	11.78

**Table 7:** Range analysis of the unconfined compressive strength by taking the 7 d value as reference.

Extremum Difference Analysis	Factor			
	Cement Content	RCA Replacement Rate	DS Replacement Rate	FA Replacement Rate
Mean 1	4.038	5.221	5.443	8.022
Mean 2	5.560	5.736	6.137	6.160
Mean 3	7.082	7.309	6.422	6.106
Mean 4	8.278	6.691	6.956	4.669
Range	4.240	2.088	1.513	3.353

**Table 8:** The variance analysis results of 7 d unconfined compressive strength.

Factor	Cement Content	RCA Replacement Rate	DS Replacement Rate	FA Replacement Rate	Error
Square of deviance	40.693	10.557	4.768	22.665	1.259
Degree of freedom	3	3	3	3	15
Estimate of variance	13.654	3.519	1.589	7.555	0.420
F_0.01_	5.417	5.417	5.417	5.417	—
F_0.05_	3.287	3.287	3.287	3.287	—
F	32.313	8.383	3.786	17.997	—

**Table 9:** Range analysis results of the 90 d splitting tensile strength.

Group Number	Factor				Targets of Test
	Cement Content	RCA Replacement Rate	DS Replacement Rate	FA Replacement Rate	90 d Splitting Tensile Strength/MPa
DRCSM-1	6%	0%	0%	0%	0.69
DRCSM-2	6%	25%	10%	25%	0.52
DRCSM-3	6%	50%	20%	50%	1.07
DRCSM-4	6%	75%	30%	75%	0.58
DRCSM-5	8%	0%	10%	50%	0.75
DRCSM-6	8%	25%	0%	75%	0.49
DRCSM-7	8%	50%	30%	0%	1.58
DRCSM-8	8%	75%	20%	25%	1.03
DRCSM-9	10%	0%	20%	75%	0.66
DRCSM-10	10%	25%	30%	50%	1.37
DRCSM-11	10%	50%	0%	25%	2.02
DRCSM-12	10%	75%	10%	0%	1.79
DRCSM-13	12%	0%	30%	25%	1.79
DRCSM-14	12%	25%	20%	0%	2.24
DRCSM-15	12%	50%	10%	75%	1.20
DRCSM-16	12%	75%	0%	50%	1.31
Mean 1	0.715	0.9725	1.1275	1.575	—
Mean 2	0.9625	1.155	1.065	1.34	—
Mean 3	1.46	1.4675	1.25	1.125	—
Mean 4	1.635	1.1775	1.33	0.7325	—
Range	0.92	0.495	0.265	0.8425	—

**Table 10:** Variance analysis results of the 90 d splitting tensile strength.

Factor	Cement Content	RCA Replacement Rate	DS Replacement Rate	FA Replacement Rate	Error
Square of deviance	0.830	0.175	0.087	0.465	0.049
Degree of freedom	3	3	3	3	15
Estimate of variance	0.277	0.058	0.029	0.155	0.016
F_0.01_	5.417	5.417	5.417	5.417	—
F_0.05_	3.287	3.287	3.287	3.287	—
F	17.090	3.606	1.782	9.572	—

**Table 11:** Test results of compressive resilient modulus of mixture.

Group Number	Compressive Resilient Modulus/MPa		
	7-d	28-d	90-d
DRCSM-1	1322.87	1670.31	2906.35
DRCSM-2	1349.04	1604.13	2807.23
DRCSM-3	1745.89	2298.16	4021.78
DRCSM-4	1344.48	1589.26	2669.96
DRCSM-5	1455.95	1898.61	3246.62
DRCSM-6	1299.96	1495.18	2586.66
DRCSM-7	2449.86	3233.38	5561.41
DRCSM-8	1748.99	2277.79	3872.25
DRCSM-9	1604.13	1875.55	3169.68
DRCSM-10	2061.42	2399.28	4126.77
DRCSM-11	2109.90	2724.95	4659.67
DRCSM-12	2634.21	3321.13	5745.55
DRCSM-13	2427.35	3157.77	5557.68
DRCSM-14	2653.92	3256.24	5698.42
DRCSM-15	2106.60	2712.71	4692.99
DRCSM-16	2245.18	2791.22	4856.72

**Table 12:** Range-analysis results of 90 d compressive resilient modulus.

Extremum Difference Analysis	Factor			
	Cement Content	RCA Replacement Rate	DS Replacement Rate	FA Replacement Rate
Mean 1	3101.33	3720.08	3752.35	4977.93
Mean 2	3816.73	3804.77	4123.10	4224.21
Mean 3	4425.42	4733.96	4190.53	4062.97
Mean 4	5201.45	4286.12	4478.96	3279.82
Range	2100.12	1013.88	726.61	1698.11

**Table 13:** The variance analysis results of the 90 d compressive resilient modulus.

Factor	Cement Content	RCA Replacement Rate	DS Replacement Rate	FA Replacement Rate	Error
Square of deviance	5,484,996	636,663	402,212	1,548,590	266,688
Degree of freedom	3	3	3	3	15
Estimate of variance	1,828,332	212,221	134,071	516,197	88,896
F_0.01_	5.417	5.417	5.417	5.417	—
F_0.05_	3.287	3.287	3.287	3.287	—
F	20.567	2.387	1.508	5.807	—

**Table 14:** Average range of 90 d compressive resilient modulus of mixture under different cement contents/%.

Cement Content	6%	8%	10%	12%
90 d compressive resilient modulus	3101.25	3816.75	4425.75	5201.50

**Table 15:** Proportion of different pore diameters in the specimen.

Group Number	Harmless Pores (<20 nm)	Less Harmful Pores (~20 nm–50 nm)	Harmful Pores (~50 nm–200 nm)	More Harmful Pores (>200 nm)
DS30FA0	14.04%	13.75%	27.94%	44.27%
DS30FA25	14.17%	15.61%	33.69%	36.53%
DS30FA75	16.97%	16.07%	33.70%	33.27%
DS0FA50	13.70%	9.37%	15.44%	61.49%
DS10FA50	11.49%	14.57%	32.46%	41.48%
DS30FA50	26.17%	24.03%	16.16%	33.63%

**Table 16:** Calculation results of life-cycle carbon emissions of 1 m^3^ CSM.

Group Number	$\gamma_{c}$	$\gamma_{HD}$	C (kg)	$g_{RC}$	W	$x_{c}$	$C_{5}$
NCA	1	0.85	126.9	1	199.2	11.3	2.9
RCSM-25			126.9	1.125	199.2	14.3	3.7
RCSM-50			126.9	1.25	199.2	17.7	4.6
RCSM-75			126.9	1.375	199.2	21.4	5.6
RCSM-100			126.9	1.5	199.2	25.4	6.6

Note: RCSM-25, RCSM-50, RCSM-75, and RCSM-100 represent the recycled aggregate replacement rate of CSM of 25%, 50%, 75%, and 100%, respectively; NCA represents the conventional CSM.

## Data Availability

Data analyzed or generated during the research period already exists in the main text, for the use of experts and scholars.

## References

[1] Cong Z., Zheng N., Yan H. (2011). Comprehensive evaluation method for road performance of semi-rigid base materials. J. Transp. Eng..

[2] (2006). Technical Specification for Highway Subgrade Construction.

[3] Guo G., Zhang F. (2018). Study on the ratio of cement stabilized gravel aeolian sand base based on unconfined compressive strength. Sci. Technol. Eng..

[4] Guo G., Zhang Y., Du S. (2017). Experimental Study on Shear Strength of Cement Stabilized Aeolian Sand Base. Sci. Technol. Eng..

[5] Zhang C., Ding J., Guo J. (2002). The application of waste cement concrete recycled aggregate in semi-rigid base. J. Chang’an Univ. (Nat. Sci. Ed.).

[6] Sun J., Jiang H., Liu S. (2009). Effect of recycled concrete aggregate on the performance of cement stabilized macadam and its engineering application. Concr. Cem. Prod..

[7] Yang J., Li X., Chen J. (2014). Experimental study on waste concrete used as cement stabilized base. J. Environ. Eng..

[8] Hu Z., Jia Z., Zhang W. (2016). Experimental Study and Engineering Application of Cement Stabilized Recycled Aggregate Base. Constr. Technol..

[9] Li Q., Wang Z., Li Y., Shang J. (2018). Cold recycling of lime-fly ash stabilized macadam mixtures as pavement bases and subbases. Constr. Build. Mater..

[10] Miao Y., Yu W., Hou Y., Liu C., Wang L. (2018). Influences of Clay Brick Particles on the Performance of Cement Stabilized Recycled Aggregate as Pavement Base. Sustainability.

[11] Disfani M., Arulrajah A., Haghighi H., Mohammadinia A., Horpibulsuk S. (2014). Flexural beam fatigue strength evaluation of crushed brick as a supplementary material in cement stabilized recycled concrete aggregates. Constr. Build. Mater..

[12] Ji X., Jiang Y., Liu Y. (2016). Evaluation of the mechanical behaviors of cement-stabilized cold recycled mixtures produced by vertical vibration compaction method. Mater. Struct..

[13] Corradini A., Cerni G., Porceddu P.R. (2021). Comparative study on resilient modulus of natural and post-quake recycled aggregates in bound and unbound pavement subbase applications. Constr. Build. Mater..

[14] Lyu X., Lyu W., Li Y. (2018). Experimental Study on Cement Stabilized Construction Waste Recycled Road Subbase. Highw. Transp. Technol. (Appl. Technol. Ed.).

[15] Peng L. (2017). Study on the Road Performance of Recycled Aggregate in Cement Stabilized Macadam Base. Master’s Thesis.

[16] Zhao B. (2020). Effect of Waste Concrete on Fatigue Properties of Cement Stabilized Soil. Master’s Thesis.

[17] Hu H., Sun Y. (2009). Experimental study on mix proportion design and strength law of recycled aggregate cement stabilized macadam. J. Hefei Univ. Technol. (Nat. Sci. Ed.).

[18] Zou G., Wang H., Fang S. (2018). Research on road performance of cement stabilized recycled aggregate. Highw. Eng..

[19] Yu C. (2020). Research on the Application of Cement Stabilized Recycled Aggregate in Road Base. Master’s Thesis.

[20] Lan X., Zhang X., Hao Z., Wang Y. (2022). Strength and shrinkage properties of cement stabilized macadam bases incorporating 0–2.36 millimetre recycled fine aggregate. Case Stud. Constr. Mater..

[21] Arulrajah A., Disfani M., Haghighi H., Mohammadinia A., Horpibulsuk S. (2015). Modulus of rupture evaluation of cement stabilized recycled glass/recycled concrete aggregate blends. Constr. Build. Mater..

[22] Cui Q., Liu G., Zhang Z., Fang Y., Gu X. (2023). Experimental Investigation on the Strength and Microscopic Properties of Cement-Stabilized Aeolian Sand. Buildings.

[23] Lopez-Querol S., Arias-Trujillo J., GM-Elipe M., Matias-Sanchez A., Cantero B. (2017). Improvement of the bearing capacity of confined and unconfined cement-stabilized aeolian sand. Constr. Build. Mater..

[24] Bai L., Yang Z., Wu Y., Anbarlouie M., Pan Z. (2023). Stabilization of Aeolian Sand for Pavement Subbase Applications Using Alkali-Activated Fly Ash and Slag. Minerals.

[25] Jing H., Zhang J., Gao M., Liu Q., Wolowiec-Korecka E. (2021). Base performances of cement-stabilized magnesium slag-aeolian sand mixture. Acta Montan. Slovaca.

[26] Netterberg F., Elsmere D. (2015). Untreated aeolian sand base course for low-volume road proven by 50-year old road experiment. J. South Afr. Inst. Civ. Eng..

[27] Song L., Li B. (2020). Effect of Aeolian Sand Content on Mechanical Properties and Durability of Cement Stabilized Graded Macadam. Silic. Bull..

[28] Ma S., Chang J., Wei L. (2004). Orthogonal Experimental Study on Cement Stabilized Aeolian Sand Macadam Base. J. Chongqing Jiaotong Univ..

[29] Xiao J., Li A., Ding T. (2016). Life cycle CO_2_ emission assessment of recycled concrete. J. Southeast Univ. (Nat. Sci. Ed.).

[30] Marinković S., Radonjanin V., Malešev M., Ignjatović I. (2010). Comparative environmental assessment of natural and recycled aggregate concrete. Waste Manag..

[31] (2015). Technical Guidelines for Construction of Highway Roadbases.

[32] (2009). Test Methods of Materials Stabilized with Inorganic Binders for Highway Engineering.

[33] Gong X., Nie Z., Wang Z. (2012). Life cycle energy consumption and carbon dioxide emission of residential building designs in Beijing. J. Ind. Ecol..

[34] Xiao J., Lei B. (2008). Carbonation model and structural durability design for recycled concrete. J. Archit. Civ. Eng..

[35] Li C. (2009). Study on the Carbonation Performance of Concrete Mixed with Mineral Admixtures. Master’s Thesis.

[36] Lee S., Park W., Lee H. (2013). Life cycle CO_2_ assessment method for concrete using CO_2_ balance and suggestion to decrease CO_2_ of concrete in South-Korean apartment. Energy Build..

[37] Wu Z. (1979). Discussion on the recent development direction of concrete science and technology. J. Chin. Ceram. Soc..

